# Metabolic syndrome in Indian patients with rheumatoid arthritis and its correlation with disease activity

**DOI:** 10.1186/ar3667

**Published:** 2012-02-09

**Authors:** Aman Sharma, Nilesh Bhilave, Kusum Sharma, Indu Varma

**Affiliations:** 1 Internal Medicine, PGIMER, Chandigarh, India,160012; 2 Medical Microbiology, PGIMER, Chandigarh, India,160012; 3 Biochemistry, PGIMER, Chandigarh, India, 160012

## Background

Increased prevalence of metabolic syndromein rheumatoid arthritis (RA) has been reported from American and European populations but it has not been studied in Indian patients with RA.

## Objectives

The main objective of our study was to assess the prevalence of the metabolic syndrome in Asian-Indian patients with rheumatoid arthritis and also to studyits correlation with disease activity.

## Methods

This was a prospective case control study in which 114 patients diagnosed to have rheumatoid arthritis of more than 1 year duration and 114 healthy age (± 5 years) and sex matched controls were included. Height, weight, body mass index, blood pressure and waist circumference of the patients were measured at the enrolment visit. Venous samples were taken after eight hours of overnight fasting for the estimation of serum cholesterol, triglycerides and plasma glucose levels. Metabolic syndrome was diagnosed according to Adult Treatment Panel III criteria [1] and the consensus definition of the metabolic syndrome for adult Asian patients [2].The disease activity was assessed by DAS 28.

## Results

The mean age of patients with RA and control group was 44.8 and 43.2 years (p <0.36) respectively. The mean duration of RA was 6.5 years. Though the mean BMI was similar in both the groups(25.5 and 24.2), there was a statistically highly significant difference in mean waist circumference(92.1 cm and 81.2 cm, p < 0.001) and diastolic blood pressure(80.5 and 75.3 mm Hg, p < 0.001) in patients with RA as compared to controls. Metabolic syndrome was present in 36 patients and 17 controls (p < 0.05) according to the Adult Treatment Panel III criteria and in 40 patients and 18 controls(p < 0.01) according to the consensus definition of the metabolic syndrome for adult Asian patients. There was no significant correlation between the metabolic syndrome and disease activity as measured by DAS-28 using both the criteria.

## Conclusions

Indian patients with RA have increased prevalence of metabolic syndrome as compared to their age and sex matched healthy controls, but there is no significant correlation between metabolic syndrome and disease activity.
